# An integrated system for genetic analysis

**DOI:** 10.1186/1471-2105-7-210

**Published:** 2006-04-19

**Authors:** Simon Fiddy, David Cattermole, Dong Xie, Xiao Yuan Duan, Richard Mott

**Affiliations:** 1 Wellcome Trust Centre for Human Genetics, Roosevelt Drive, Oxford OX3 7BN, UK

## Abstract

**Background:**

Large-scale genetic mapping projects require data management systems that can handle complex phenotypes and detect and correct high-throughput genotyping errors, yet are easy to use.

**Description:**

We have developed an Integrated Genotyping System (IGS) to meet this need. IGS securely stores, edits and analyses genotype and phenotype data. It stores information about DNA samples, plates, primers, markers and genotypes generated by a genotyping laboratory. Data are structured so that statistical genetic analysis of both case-control and pedigree data is straightforward.

**Conclusion:**

IGS can model complex phenotypes and contain genotypes from whole genome association studies. The database makes it possible to integrate genetic analysis with data curation. The IGS web site  contains further information.

## Background

Advances in high-throughput genotyping technology mean that it is now possible to carry out very large-scale studies to identify the genetic basis of complex disease in human and animal models. Whole genome association studies are now being carried out in humans and rodents, in which hundreds of thousands of genetic markers are typed on thousands of samples, imposing new demands on data management systems. The challenge is not merely how to manage the vastly increased data volume but also how to integrate data collection, quality control and analysis. Up to now, genetic mapping studies have proceeded linearly from data collection through data cleaning to data analysis, which is often carried out by scientists who will have had no direct involvement with the prior phases of the project. Available databases reflect the work flow pattern so that rarely are raw data available at the stage of analysis. However, error detection is often only possible at the last stage of the project, when, for example, data from different family members across a whole genome can reveal subtle departures from expected patterns. Such problems can be dealt with by simply deleting problematic data with consequent loss in power for the study, or, in small projects, it may be possible to trace the error back to some point in data collection where it may be corrected. The importance of error detection has only recently been acknowledged, but it is clear that some study designs are extremely sensitive to error: for example, undetected error rates of about 1% can reduce the power of linkage studies of quantitative traits by 50% [1]. Ideally it should be possible to integrate the components of the genetic mapping project so that there is a seamless combination of data collection and analysis.

Many available products separate the process of data editing from that of data collection. For example, the commercially available software packages BC|GENE from Biocomputing Ltd [2]. and the Scierra Genotyping Laboratory Workflow System from CimSoft [3] do not allow the user to detect and correct errors from the raw data. The same applies to the products of collaboration with academic institution GIDS [4] and SNPP [5]. Many other laboratories have developed their own systems designed for a particular workflow and while some systems do incorporate Mendelian error checking of genetic data, none have the flexibility that we needed. Therefore we set out to design and implement a system that satisfies the following requirements: storage and access to raw data, both genotypes and phenotypes; inclusion of quality control tools within the database and functions that allow users to edit data; flexible definitions of phenotypes to allow the inclusion of multivariate data; genetic analysis programmes integrated into the database; download formats compatible with genetic analysis programmes. The result is the Integrated Genotyping System (IGS), which we use to support many of the projects to map genes related to complex disease at the Wellcome Trust Centre for Human Genetics.

## Construction and content

IGS integrates data collection and analysis with a relational database. There are four core features that make this possible: (i) development of interfaces between the genotyping platforms and the database, so that all data are directly uploaded; (ii) the ability to store all types of phenotypic information; (iii) the inclusion of tools for editing all data types so that all values can be regarded as provisional; (iv) analysis can be carried out an any stage.

**Figure 1:**
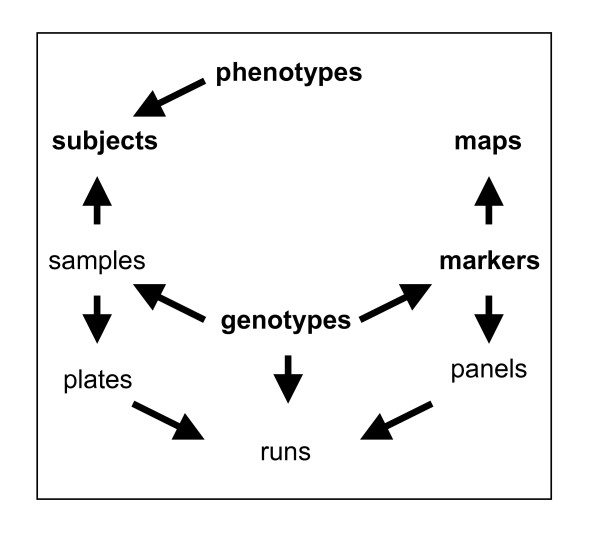
Conceptual view of a genetic mapping experiment in IGS. The arrows represent the direction of work that takes place in the experiment. For example, phenotypes are collected on subjects, genotypes from samples that are taken from subjects.

The IGS model of a genetic mapping project is shown in Figure 1. The objects in bold (**subjects, phenotypes, maps, markers **and **genotypes**) are of primary scientific interest, in that the aim of the project is to identify statistically significant associations between phenotypes and genotypes across a set of subjects and genetic map of markers. The other objects are laboratory entities which should be modeled in order to carry out the experimental work and to ensure fidelity in the data. The arrows indicate the relationship "belongs to". For example, samples and phenotypes belong to subjects, and genotypes belong to samples and to markers.

IGS supports multiple independent studies in the same database and manages its own database of users and user roles. IGS stores information about the subjects in each study, the subjects' phenotypes, the genetic markers and the genotypes. It models phenotypes of arbitrary complexity, including multivariate and quantitative traits, as well as laboratory entities such as DNA samples, primer sequences and microtitre plates. Data access in IGS is via a secure web interface for interactive work or via a PERL console for scripting. IGS has a sophisticated yet intuitive interface to extract and analyse data, and extensive quality control and data editing facilities. Analyses are linked directly to the system, or data can be downloaded in formats suitable for analysis, such as pedigree format files.

### IGS contains the following data types

#### users, groups and projects

IGS manages its own user accounts and passwords. Each IGS *user *has a role which controls the IGS web pages they can access, and hence what they can do (e.g. read access only). Each user belongs to one or more IGS *groups*, which are collections of IGS *projects*. A project comprises an autonomous collection of subjects, samples, plates, markers and genotypes. Projects do not share data.

#### subjects and samples

In IGS a *subject *is an individual within a project, and has an identifier (e.g. a barcode) unique within that project. Family relationships are handled by specifying parents and monozygotic siblings. Subjects carry basic information about their gender, date of birth and of death. IGS distinguishes between a subject and its associated DNA *samples*; a subject can have multiple samples. This mechanism allows the definition of aliases, and helps track the source DNA of genotypes accurately. Sample identifiers are unique within a project.

In IGS subjects are grouped by family, where applicable, or by defining *subject groups *which are used for repeated extraction and analysis of data related to the same subjects. Subject groups can either be defined by uploading a file of subject names or created interactively, based on criteria such as family membership or membership of existing groups. Samples can also be grouped in a similar way.

#### phenotypes and covariates

In IGS a *phenotype *can be *simple*, i.e. a single observation such as disease status, or *complex*, a vector of named phenotype elements. Each element has a data type (free text, URI, date, number or from a controlled vocabulary, and is classified as a phenotype or a *covariate *(e.g. the date, or the experimental conditions). Phenotypes can also be repeat-valued (e.g. a time series). IGS Phenotypes are ideal for storing questionnaire data, as IGS automatically generates an appropriate form interface for data entry and editing. Raw data such as images can be treated as IGS phenotypes by storing the raw data files in a web-accessible directory and using the corresponding URIs as IGS phenotype elements. This mechanism also links subjects to external web resources. Phenotypes are defined through a web-based interface, or by uploading a phenotype definition file. A complex phenotype can have an arbitrary number and variety of elements, but must be fully defined before data is uploaded.

#### plates

In IGS a *plate *is a named collection of 96 or 384 samples with their well locations. Multiple samples (for pooling) and markers (for multiplexing) can be associated with the same well.

#### markers

IGS recognizes three types of *marker*, SNPs, microsatellites and generic markers. Microsatellites contain information about the repeat unit and the left and right PCR primer sequences, while SNPs store information about the possible alleles and require an extension primer sequence. Any number of URI links can be associated with a marker (e.g. to point to the corresponding location in a genome browser). Currently IGS only stores the species and chromosome for a marker, as the marker's genetic location varies depending on the genetic map used. The centi-Morgan map positions for markers are specified in an externally created map file for upload when required.

#### genotypes

IGS distinguishes between a *genotype observation*, measured on a marker and a sample, and a *final genotype*, attached to a marker and a subject. An unlimited number of genotype observations can be associated with each final genotype. The final genotype is initially automatically assigned as the first uploaded genotype for a given marker and subject combination. Upon subsequent genotypes being uploaded, IGS will automatically detect where there are inconsistent genotype observations. IGS then defines the final genotype as the most frequent observation, but flags this inconsistency for correction by the user. A genotype observation contains optional information about the plate and well location of the sample. It also has data on the genotyping call method (i.e. the type of genotyping instrumentation, such as ABI3700, MegaBace, Sequenom, Illumina) and a quality score. Only final genotypes are exported for statistical analysis, not the genotype observations. IGS has a quality control tool to identify and correct final genotypes with inconsistent genotype observations.

## Utility and discussion

IGS is designed to house and analyze large genetic mapping projects. At present, the largest projects using IGS is a study to map quantitative trait loci in outbred mice [6]. In outline, over 100 phenotype observations have been made on 2000 mice. The phenotypes include behavioural, physiological and biochemical measurements. In some cases the phenotypes include images (from tracking cameras, for instance) and other binary data, which are tied to the database by storing their URIs. The project work is distributed over a number of sites, so the ability to access the database securely over the web is a major advantage. Approximately 40 million SNP genotypes for the project have been uploaded and IGS is key to the curation and analysis of this dataset.

### Data upload and download

Data can be uploaded into an IGS project from tabular files prepared according to pre-defined IGS templates. There are separate templates for subjects, samples, markers, phenotype definitions, phenotype values, plates and genotype observations. Genotype upload varies according to the genotyping platform. For Sequenom-generated genotypes, data are fetched automatically from the Sequenom system's own Oracle tables, and progress can be monitored and controlled via a specialized web page in IGS. Illumina genotypes are first converted into IGS upload format using a PERL-CGI web interface and then uploaded into IGS.

**Figure 2:**
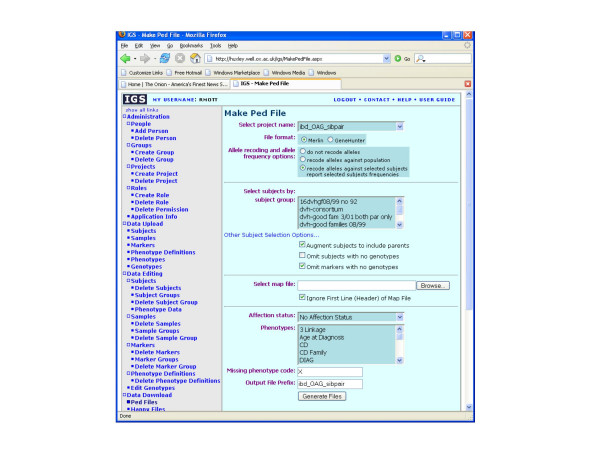
The IGS web interface to download Ped files. The user selects subjects and markers by choosing a subject group and a map file. Phenotypes are selected by name. Options controlling the output format are available as checkboxes. The left margin allows the user to navigate between the IGS web pages. Context-sensitive assistance is obtained by clicking on the top-right HELP button.

Data can be downloaded in a variety of formats. Users can make *table extracts *of all the data in a project. Tables are presented in a searchable and filterable environment, and are downloadable as Excel spreadsheets, csv, xml or tab-delimited text files. Certain table extract formats correspond exactly with table upload format, so that it is possible to extract data, perform bulk edits and then re-import it. Most statistical analysis software read pedigree format files, and IGS has a sophisticated downloader for pedigree format files (Figure 2). It supports two variants: in GeneHunter format [7], all the subjects are recoded as pairs of integers representing the family and individual id, and all alleles recoded as integers. In Merlin format [8] the subject names are left unaltered and the alleles are not recoded by default. IGS also creates the associated .dat (and in the case of Merlin, .map and .alleles) files. The user can specify which genotypes are to be extracted from a list of subject groups and pedigrees, and which markers to use by uploading a map file of marker names and locations. The user can also select any phenotype element as the affection status and append any additional phenotype elements. Once the pedigree files have been created they can be downloaded for analysis, or processed from a menu of standard analyses via a web service, implemented in PERL CGI running on a Linux server. Available applications include Merlin[8], QTDT[9], Phase [10] and Transmit [11]. To add an application to the menu one first registers it in the CGI script, and then writes a PERL wrapper to handle input file conversion if the application does not read pedigree files directly.

### Data analysis and quality control checks

IGS objects can be created, edited and deleted interactively from specialised web pages. Genotypes are edited by modifying the value of the final genotype for a subject and marker. A page displays the raw information from the genotyping machine, the assigned values of the genotypes and, when available, conflicting information from the same subject. The user is then able to make a decision as to the final value of the genotype that will be used in genetic analyses or downloaded from the database. In some instances bulk editing is best accomplished by making a table extract, editing it outside of IGS and then reimporting the data.

IGS tests for **association **between a marker and a trait, using analysis of variance for quantitative and the chi-squared test for categorical traits. Sex effects may be taken into account. Further analyses using external software (e.g. Merlin) are integrated as web services. Quality checks are implemented in IGS: a pedigree check, a test for Hardy Weinberg equilibrium and an allele frequency test. The **pedigree check **identifies offspring alleles that do not occur in the parents. Summaries of pedigree error counts, categorized by subject and marker are provided to identify subjects or markers with many errors, which may indicate errors in the stated family relationships, sample labeling, or an unreliable marker. Sporadic genotype problems can be edited by following a link to the genotype editor page. The **Hardy-Weinberg test **finds markers with unlikely genotype frequencies in case-control studies. The **allele frequency test **compares allele frequencies in a subject group with those in the remainder of the population.

These tools identify subjects and markers with abnormally high error rates. Such data are either deleted from the system, or a special *quality control group *of subjects/markers may be defined that acts as a filter when data are exported as ped files for analysis – the corresponding genotypes are set to be missing in the output, but the genotypes are not deleted from the database. Although IGS does not maintain an audit trail of changes, it records the date when a genotype was last modified.

### Implementation

We implemented IGS using Microsoft SQL Server 2000 (Enterprise Edition) as the backend database server and Microsoft Visual Studio .NET as the development tool. The web interface is mainly an ASP.NET application running on top of IIS Server 6.0 with .NET Framework 1.1 running on Windows Server 2003 Enterprise Edition. It is theoretically possible to run the web interface on major Linux servers with the Mono framework and apache web server.

The IGS database comprises 70 tables and over 310 stored procedures. It can be setup on most of the modern relational databases as long as the database supports stored procedures. Some recoding is necessary to reflect syntax differences in stored procedures. Considering the large scale data IGS is handling, an enterprise level product should be used to provide the reliability, availability, and scalability, e.g. Oracle, IBM DB2, and Microsoft SQL. Open source database like MySQL can be used as well since the most recent version of MySQL (5.0, released at the end of 2005) added the stored procedure ability.

All data access is through three software layers: The Data Access Layer (DAL), written in C#, controls all access to the underlying database tables and stored procedures. Conceptually IGS deals with objects such as users, subjects, markers, genotypes etc, that are modeled in the Application Logic Layer (ALL), also written in C#. The Web Access Layer (WAL), written in VB.Net and ASP.Net controls user interaction. Manipulation of IGS objects is accomplished by calling the corresponding object methods in the ALL. For reasons of efficiency, bulk data load/dump bypass the object models to call stored procedures directly.

### Web access

Interactive access to IGS is via 97 ASP.Net web pages and popup windows. Related pages, such as Data upload, Data Download, Data Analysis are grouped together, although certain pages are accessible from different points in the hierarchy. The IGS login page uses https to transmit passwords securely; the remainder of the site is accessible over our intranet via http and externally over the Internet by https. Once logged in, the user navigates through IGS using links on the left margin of the IGS web pages. Access to pages is restricted according to the user's IGS role. Context-sensitive help is available on each page. IGS uses a style sheet, which ensures it has the same functionality and appearance on different web browsers (Netscape, Internet Explorer and Firefox) and platforms (Windows PC, Mac X, and Linux). An example page is shown in Figure 2.

### Command-line PERL Console

IGS has a PERL console (implemented in the PERL module IGS.pm) to script the extraction of tables and ped files for automated analysis. Internally the console uses the same http and https protocols (i.e. not ODBC) and functions in the same way as the secure web interface, requiring logins and passwords. The functions available through the PERL console are identical to the data extraction functions available through the web interface; the module's purpose is to enable automated and scripted downloads.

### Performance

The production version of IGS runs on a DELL PowerEdge 6650 four-way 1.4 GHz processor server with 12 GB RAM and a 700 GB RAID. Both the SQL-Server engine and the Web Server are supported on the same machine, although the system will also work with the database and interface on separate machines. Currently the production system supports over 40 projects and 50 million genotypes. Most queries to the system take a few seconds, although uploading or extracting very large datasets takes several minutes. We expect the volume of data to grow rapidly over the next year, currently with over a billion genotypes in the production version IGS, housed on a larger server.

### Development

Future IGS development will focus on integrating new genotyping platforms, on scaling up to handle extremely large datasets, and on integrating sequence annotation, genomics and data analyses. Large-scale association studies are now underway comprising thousands of subjects and 650,000 SNPs, producing billions of genotypes. Because IGS uses industry-standard components, in principle it should be capable of handling very large volumes of data, provided sufficiently powerful hardware is available (for example a RAID with large numbers of high-speed spindles). We are actively investigating this issue, and may streamline the table structure and stored procedures to speed up genotype data access.

Integrating annotation data is more straightforward and need not involve much change to the underlying IGS codebase, but rather the writing of web services. A key requirement is to ensure that IGS entities such as SNPs are mapped onto external public databases, so that one may extract genome annotations in regions with significant genetic association. Since IGS already links markers to genome browsers by URIs, the mechanism to do this is already in place.

## Conclusion

IGS is used at our Centre for the manipulation of large volumes of genotype and phenotype data. It satisfies most of our requirements: it holds data in a secure manner, is securely accessible across the web, and permits users to perform data curation tasks with a small number of mouse clicks. Close contact with users has ensured their requests are implemented quickly. The main advantages of IGS over competing systems are that it can model very complex phenotypes and that it is now possible to integrate genetic analysis with data curation.

## Availability and requirements

The IGS web site is . This contains further information, including a PDF format IGS user guide  and a web (https) accessible demonstration version of IGS. A copy of the IGS runtime and a script to generate the database schema and stored procedures can be downloaded from on the IGS website. IGS is also available to commercial groups, who should contact Richard Mott rmott@well.ox.ac.uk. IGS requires a license for Microsoft SQL-SERVER 2000.

## Authors' contributions

Simon Fiddy, David Cattermole, Dong Xie and Richard Mott were responsible for the design and programming of the database. Xiao Yuan Duan contributed to the software and development of the application.
